# A Glial Interplay Regulates Junctional Architecture at the Blood–Brain Barrier of *Drosophila*

**DOI:** 10.1002/glia.70256

**Published:** 2026-11-02

**Authors:** Esteban G. Contreras, Elke Naffin, Miguel Collins‐Ramírez, Nina V. Surina, Marco Rothermel, Jennifer Salazar‐Tirado, Stefanie Schirmeier, Christian Klämbt

**Affiliations:** ^1^ Departamento de Biología Celular, Facultad de Ciencias Biológicas Universidad de Concepción Concepción Chile; ^2^ Group for the Study of Developmental Processes (GDeP), Department of Cell Biology, Faculty of Biological Sciences Universidad de Concepción Concepción Chile; ^3^ Institute of Neuro‐ and Behavioral Biology University of Münster, Multiscale Imaging Center Münster Germany; ^4^ Zoology and Animal Physiology, Faculty of Biology Technische Universität Dresden Dresden Germany

**Keywords:** blood–brain barrier, cell ablation, perineurial glia, septate junctions, subperineurial glia

## Abstract

Neurons require a tightly controlled ion and metabolite homeostasis, which is maintained by an effective blood–brain barrier (BBB). Since the integrity of the BBB is essential for brain function, its maintenance during development represents a significant challenge. The *Drosophila* BBB is formed by two glial layers. Subperineurial glia (SPG) establish pleated septate junctions (pSJs) to generate the primary diffusion barrier. Abutting the SPG lies the mitotically active perineurial glia (PG). Only a small number of PG cells are specified during embryogenesis. The FGF8‐like signal Pyramus presented by the SPG subsequently triggers proliferation of the PG, which ultimately covers the entire nervous system. To investigate the role of PG in the development and physiology of the BBB, we genetically ablated them. In these animals, glucose influx into SPG is unchanged following acute glucose perfusion. However, adult PG‐ablated flies show increased sensitivity to glucose‐only diet, indicating that while baseline carbohydrate transport can be maintained in the absence of most PG, the system fails under metabolic stress. SPG morphology and pSJ organization are largely preserved during larval stages upon PG ablation. In contrast, in PG‐ablated pupae, *moody* expression is impaired in SPG and pSJs progressively split, producing ectopic junctional strands. Together, these results reveal the necessity of PG for the maturation and function of SPG, as well as the mutual interdependence between the two BBB glial layers.

## Graphical abstract

- *Drosophila* perineurial glia are required for septate junction formation at the blood–brain barrier.
- Glucose transport across the blood–brain barrier is maintained in the absence of perineurial glia.
- Perineurial glia ablation increases glucose‐only diet sensitivity.

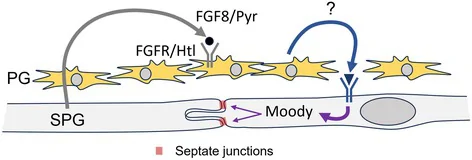

## 1 Introduction

The blood–brain barrier (BBB) of the nervous system is a highly specialized and dynamic interface that safeguards the central nervous system by regulating the exchange of ions, metabolites, and cells between the circulation and the neural tissue. By tightly controlling brain permeability, the BBB preserves neural homeostasis and protects against toxins and pathogens (Daneman and Prat 2015; Gastfriend and Daneman 2026). Rather than functioning as a simple physical barrier, the BBB is a complex, multicellular structure that exerts intricate gatekeeper functions. During development, the establishment of BBB properties requires the coordinated differentiation and spatial organization of multiple cell types (Langen et al. 2019). Disruption of this coordination can compromise barrier integrity, leading to altered brain physiology and increased susceptibility to neurological disease. Understanding how distinct cellular populations integrate developmental cues to build and sustain a functional BBB is therefore essential for elucidating both normal brain development and the mechanisms underlying barrier dysfunction.

Some invertebrate taxa, including arthropods, possess a specialized glial barrier that separates neural tissue from the circulating hemolymph (Dunton et al. 2021; Paredes‐González et al. 2025; Funke et al. 2026). In *Drosophila melanogaster* , this BBB (also referred to as the hemolymph‐brain barrier) is constituted by two distinct glial populations, the perineurial (PG) and the subperineurial glia (SPG), in conjunction with a dense extracellular matrix known as the neural lamella (Figure 1A) (Hindle and Bainton 2014; Limmer et al. 2014; Schirmeier and Klämbt 2015; Contreras and Sierralta 2022; Contreras and Klämbt 2023).

**FIGURE 1:**
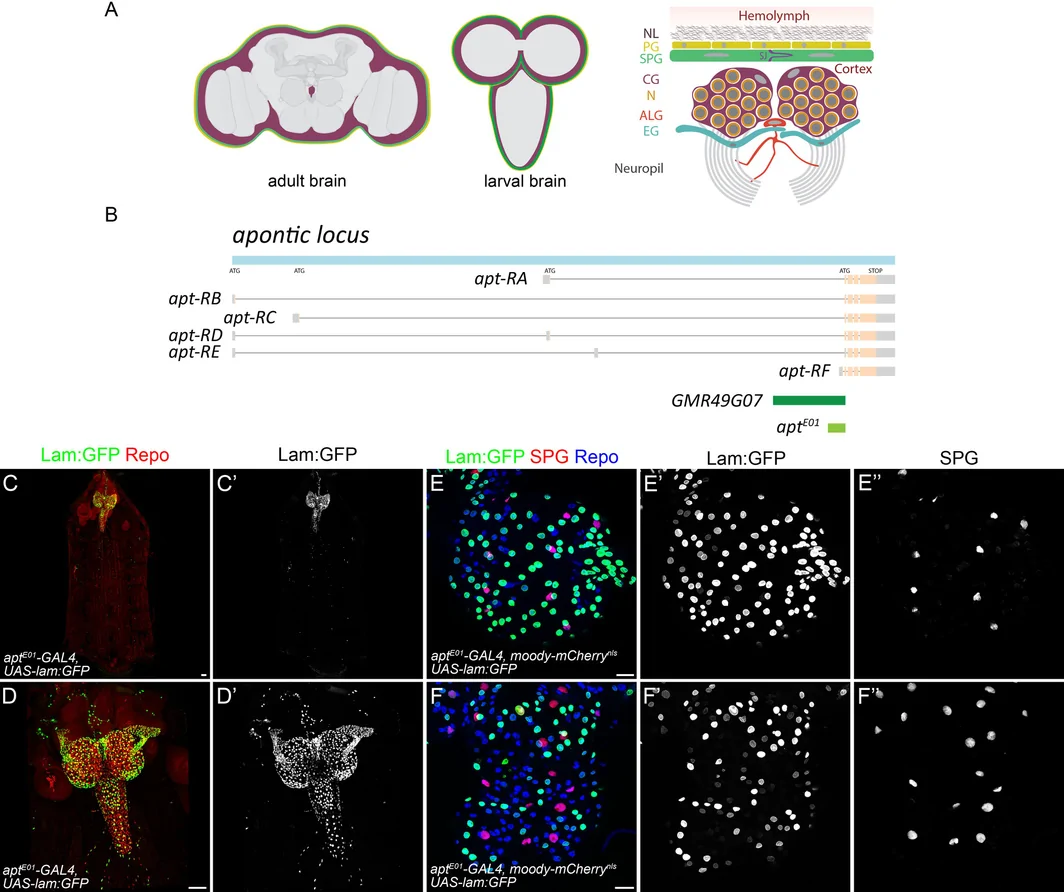
Expression pattern of the *apt* ^*E01*^ *‐GAL4* driver. (A) Schematic representation of the adult and larval *Drosophila* brain. The blood–brain barrier (BBB) is formed by the subperineurial, and the perineurial glia, and the neural lamella. ALG, astrocyte‐like glia; CG, cortex glia; EG, ensheathing glia, N, neuron; NL, neural lamella; SPG, subperineurial glia; PG, perineurial glia. (B) Schematic view of the *apontic* (*apt*) *locus* with all mRNA isoforms and the location of the *GMR49G07* and the *apt* ^*E01*^ enhancer. (C–F′) Expression pattern of the *apt* ^*E01*^ *‐GAL4* driver. (C–D′) Larval fillet of an animal of the genotype *apt* ^*E01*^ *‐GAL4 UAS‐Lam:GFP*. GFP staining is shown in green (C′–D′ gray) the pan‐glial nuclear marker Repo is shown in red. (E–F′) Immunofluorescence of a (E) brain lobe and (F) vnc of a larva with the genotype *apt* ^*E01*^ *‐GAL4, UAS‐Lam:GFP*; *moody‐mCherry* ^*nls*^. GFP staining is in green (E′–F′ gray), Repo is in blue, the nuclear SPG marker mCherry is in red. Scale bars are (C–D′) 100 μm and (E–F″) 20 μm.

The large and flat SPG cells are formed during late embryonic development and establish the primary diffusion barrier by forming extensive pleated septate junctions (pSJ). These occluding cell–cell junctions ensure the tight control of paracellular and transcellular transport into the nervous system (Tepass and Hartenstein 1994; Carlson and Hilgers 1998; Carlson et al. 2000; Schwabe et al. 2005, 2017; Beckervordersandforth et al. 2008; Stork et al. 2008). The development of the SPG is in part under the control of the G‐protein coupled orphan receptor Moody. Moody acts through Gαi and the cAMP effector PKA to control cell polarity and actin dependent pSJ formation (Bainton et al. 2005; Schwabe et al. 2005; Hatan et al. 2011; Li et al. 2021; Contreras et al. 2024). During larval development, the SPG cells undergo an enormous cell growth with endoreplication to cover the increase in brain volume caused by the second wave of neurogenesis (Unhavaithaya and Orr‐Weaver 2012; Von Stetina et al. 2018; Zülbahar et al. 2018). Notably, the barrier integrity is maintained in these stages by stretching pre‐assembled pSJ (Babatz et al. 2018).

A small number of PG progenitors form during early embryonic development (von Hilchen et al. 2013). Unlike the SPG, these cells are mitotically active and divide throughout development to fully cover the entire nervous system (Pereanu et al. 2005; Awasaki et al. 2008; Stork et al. 2008; Hartenstein 2011; Avet‐Rochex et al. 2012). They form the outermost cell layer of the BBB and are in direct contact with the hemolymph.

The function of PG has not been completely elucidated. During larval development, PG participate in neuroblast mitotic reactivation by Dally‐like, a heparan sulfate proteoglycan, and Glass bottom boat, a bone morphogenetic protein (BMP) homolog (Kanai et al. 2018; Lin et al. 2025). How changes in ECM and BMP signaling elicited by the PG are transduced to neural stem cells is still elusive. In addition to their developmental function, the PG contribute to nervous system physiology. They express various carbohydrate and monocarboxylate transporters and are thus thought to play key roles in metabolic support of the nervous system (Volkenhoff et al. 2015; Weiler et al. 2017; Delgado et al. 2018; Hertenstein et al. 2021; McMullen et al. 2021; Contreras and Sierralta 2022; De Backer et al. 2025; González‐Gutiérrez et al. 2025). PG and SPG establish gap junctions that facilitate metabolic and xenobiotic fluxes but also participate in the coordination of neuroblast division (Holcroft et al. 2013; Spéder and Brand 2014; Volkenhoff et al. 2018; Zhang et al. 2018). In adulthood, PG function has been linked to alcohol and seizure sensitivity (Parkhurst et al. 2018; Weiss et al. 2022), and to the regulation of the circadian rhythm (Dubowy and Sehgal 2017; Zhang et al. 2018; Cuddapah et al. 2019). The current data suggest a close interaction between PG and SPG. Continuous communication between PG and SPG may also be required to coordinate PG cell number with SPG growth. This coordination is likely to be particularly important during periods of rapid brain growth and remodeling, such as the pupal stage of development.

In this work, we investigated this question by genetically ablating PG during development. To achieve this, we generated a novel GAL4 driver line (*apt* ^*E01*^ *‐GAL4*) that specifically targets PG without affecting other cell types and used it to induce apoptosis. We found that during larval development, SPG morphology remained intact and glucose transport across the BBB was preserved. In contrast, during pupal development, pSJs became severely disrupted due to a *moody* dependent alteration in SPG polarity. Notably, upon genetic ablation, adults survived but showed an increased sensitivity to a glucose‐only diet. Collectively, these findings demonstrate a stage‐specific requirement for PG in the maturation and maintenance of SPG barrier architecture and reveal a functional interaction between two glial cell types during BBB development.

## 2 Results

### 2.1 Developing a Tissue‐Specific Perineurial Glial Driver

To investigate the role of perineurial glia (PG) during development, it is essential to have tools that enable tissue‐specific genetic manipulation. Most of the currently available perineurial GAL4 drivers also drive expression in other cell types and tissues, including subperineurial glia (SPG) (Table 1, Figure S1, Figure S2). To overcome this limitation, we focused on the *GMR49G07* enhancer (Jenett et al. 2012; Sasse and Klämbt 2016; Arce‐Molina et al. 2020; McMullen et al. 2021), located upstream of the *apontic‐RF* isoform (*apt‐RF*) start codon, which drives expression in PG, SPG, and tracheal cells during larval development (Figure S2). We reasoned that distinct regulatory elements within this 3784 bp long enhancer region might confer cell type‐specific expression and tested several fragments for driving PG‐specific reporter gene expression. Using this strategy, we identified a 935 bp fragment, named *apt* ^*E01*^ enhancer, which was cloned upstream of a basal promoter and the *GAL4* coding sequence to generate the *apt* ^*E01*^ *‐GAL4* driver (Figure 1B).

**TABLE 1:** Expression pattern of GAL4 drivers.

Driver	Associated gene	Expression in	References
*46F‐GAL4*	*unknown*	Perineurial glia, subperineurial glia, anal pad, salivary glands, fat body (weak).	Brink et al. (2012)
*apt* ^*E01*^ *‐GAL4*	*apontic*	Perineurial glia	This work
*GMR49G07‐GAL4*	*apontic*	Perineurial glia, subperineurial glia, tracheal cells, gut (weak).	Jenett et al. (2012)
*GMR85G01‐GAL4*	*schnurri*	Perineurial glia, salivary glands, gut, tracheal cells, leg imaginal discs, wing imaginal discs.	Kremer et al. (2017)
*NP6293‐GAL4*	*Basigin*	Perineurial glia, gut, malpighian tubes, salivary glands.	Awasaki et al. (2008)
*Tret1‐GAL4*	*Tret1*	Perineurial glia, neuroblasts, gut, Malpighian tubes, fat body, wing pouch, imaginal ring cells.	Hertenstein et al. (2021)

To assess the expression pattern of *apt* ^*E01*^ *‐GAL4* line, we expressed a fluorescent nuclear reporter under UAS control (*UAS‐lam:GFP*). Localization of GFP at the nuclear lamina was detected at the outer surface of the nervous system of third instar larvae, including the brain, the ventral nerve cord (vnc), and the peripheral nervous system (Figure 1C,D′). To evaluate the specificity of this labeling, we combined *apt* ^*E01*^ *‐GAL4*‐driven GFP expression with a newly generated SPG nuclear marker (*moody‐mCherry* ^*nls*^) and immunostained for the pan‐glial marker Repo. We observed that GFP expression strictly localizes to Repo‐positive nuclei at the surface of the larval brain and the ventral nerve cord (vnc). Notably, GFP‐positive nuclei did not colocalize with mCherry‐positive nuclei, indicating that *apt* ^*E01*^ *‐GAL4* expression is restricted to PG in the larval brain (Figure 1E,F′ and Figure S3).

Next, we examined whether the *apt* ^*E01*^ *‐GAL4* driver is expressed in all PG cells. To this end, we expressed a different fluorescent protein under the control of *apt* ^*E01*^ *‐GAL4* and compared their localization to the distribution of the known PG markers Apt and the Trehalose transporter 1 (Tret1). When nuclear localized GFP (*UAS‐Stinger*) was expressed under the control of *apt* ^*E01*^ *‐GAL4*, not all PG are equally strongly labeled. 86.7% ± 0.97% (mean ± SEM) of the central brain PG expressed the *apt* ^*E01*^ *‐GAL4*. Some Tret1 positive PG in the central brain did either not express the driver or expressed only very low levels (arrowheads in Figure S3A″). Notably, PG close to the presumptive forming optic lobes express stronger levels of *apt* ^*E01*^ *‐GAL4* driver (arrowheads in Figure S3B″).

When we expressed membrane‐tagged mCherry, we observed differential expression around the central brain lobes and thoracic segments of the vnc. Interestingly, expression levels of *apt* ^*E01*^ *‐GAL4* become so low in the abdominal neuromers that no localization of CD8‐mCherry could be detected (Figure S4A′). We found that close to two thirds of the PG in the vnc were expressing the *apt* ^*E01*^ *‐GAL4* driver (67.32 ± 1.22). These results indicate that while the *apt* ^*E01*^ *‐GAL4* driver is highly specific for PG, it is not evenly expressed in the entire PG population.

### 2.2 The *apt* ^*E01*^ *‐GAL4* Driver Enables the Ablation of Most Perineurial Glial Cells

Although several functions have been attributed to PG, direct evidence supporting their contribution to BBB development remains limited. To address this, we ablated PG during development to understand their role (Figure 2A). For this purpose, we drove the expression of the pro‐apoptotic genes *reaper* (*rpr*) and *head involution defective* (*hid*) (Zhou et al. 1997) using the *apt* ^*E01*^ *‐GAL4* line.

**FIGURE 2:**
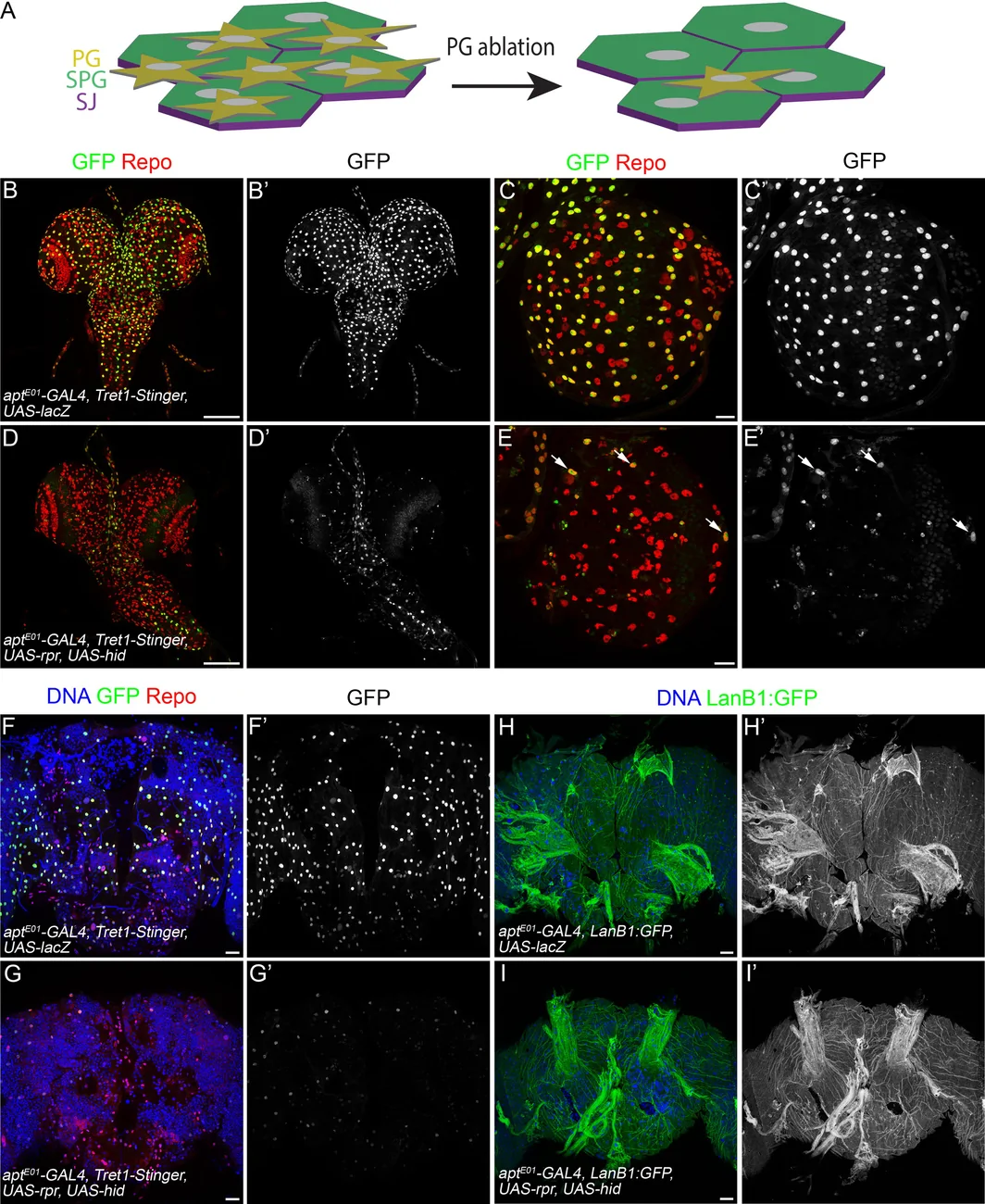
Perineurial glial ablation using the *apt* ^*E01*^ *‐GAL4* driver. (A) Diagram of the strategy of genetic ablation of perineurial glia at the BBB. SJ, septate junction; SPG, subperineurial glia; PG, perineurial glia. (B–E′) Third instar larval brains stained for Repo (red) and Stinger‐GFP localization (green, gray). (B–C′) Control larvae of the genotype *apt* ^*E01*^ *‐GAL4, Tret1‐Stinger, UAS‐lacZ*. (D–E′) Ablation of perineurial glia in larvae of the genotype *UAS‐rpr; apt* ^*E01*^ *‐GAL4, Tret1‐Stinger; UAS‐hid*. Arrows point to remaining PG nuclei. (F–G′) Adult brains stained for DAPI (blue), Repo (red), and Stinger‐GFP localization (green, gray). (H–I′) Adult brains stained for Laminin localization (green, gray), and DAPI (blue). (F, F′) Control animal of the genotype *apt* ^*E01*^ *‐GAL4, LanB1:GFP, UAS‐lacZ* (control), and (G, G′) Perineurial glia ablated fly with the genotype *UAS‐rpr; apt* ^*E01*^ *‐GAL4, LanB1:GFP; UAS‐hid*. (H–I′) Adult brains stained for Repo (red), DAPI (blue), and GFP (green and gray). Scale bars are (B–D′)100 μm, (C–I′) 20 μm.

We found that most PG of the larval CNS, labeled with the PG reporter *Tret‐1‐1‐Stinger* (Hertenstein et al. 2021), were eliminated upon the ectopic expression of *rpr* and *hid* (Figure 2B–D′, Figure S4C). A small number of *Tret1‐Stinger*‐positive glial cells persisted in the abdominal vnc and in the central brain (Figure 2B–E′), indicating that PG ablation was not completely penetrant. Ablation efficacy was further tested by analyzing the distribution of the trehalose transporter Tret1, which is normally expressed in PG and neuroblasts in the larval brain (Figure S4A). Interestingly, residual Tret1 expression can be detected in the abdominal neuromeres (Figure S4B). Consistently, when we assessed PG persistence by co‐labeling of Apt and Repo after ablation, we observed that only a few PG survive in the larval brain, whereas several PG persisted in the thoracic and abdominal segments of the vnc (Figure S5).

We next analyzed *Tret‐1‐Stinger* expression in the adult brain. Here, we also observed some surviving PG cells expressing the reporter in the BBB (Figure 2F,G′). This suggests that a subset of PG did not express (high levels of) *rpr* and *hid* and survived into adulthood. To assess BBB integrity in adults, we examined the presence of the neural lamella using a transgenic line that labels Laminin B1 (*lanB1:GFP*). The extracellular matrix surrounding the adult brain appeared intact, indicating that PG ablation during larval development did not overtly compromise neural lamella formation (Figure 2H,I′).

### 2.3 Perineurial Glial Ablation Affects Brain Development

To examine the consequences of PG ablation during development, we first analyzed growth of the larval central nervous system. The size of third instar larval brain subjected to PG ablation was not significantly different from that of control animals; however, we noted a tendency toward smaller brains following PG ablation (Figure 3A–C). This size difference was more pronounced in adult stages, where brain size was significantly reduced following larval PG ablation (12% reduction, Figure 3D–F). This demonstrates that the loss of most PG affects brain size.

**FIGURE 3:**
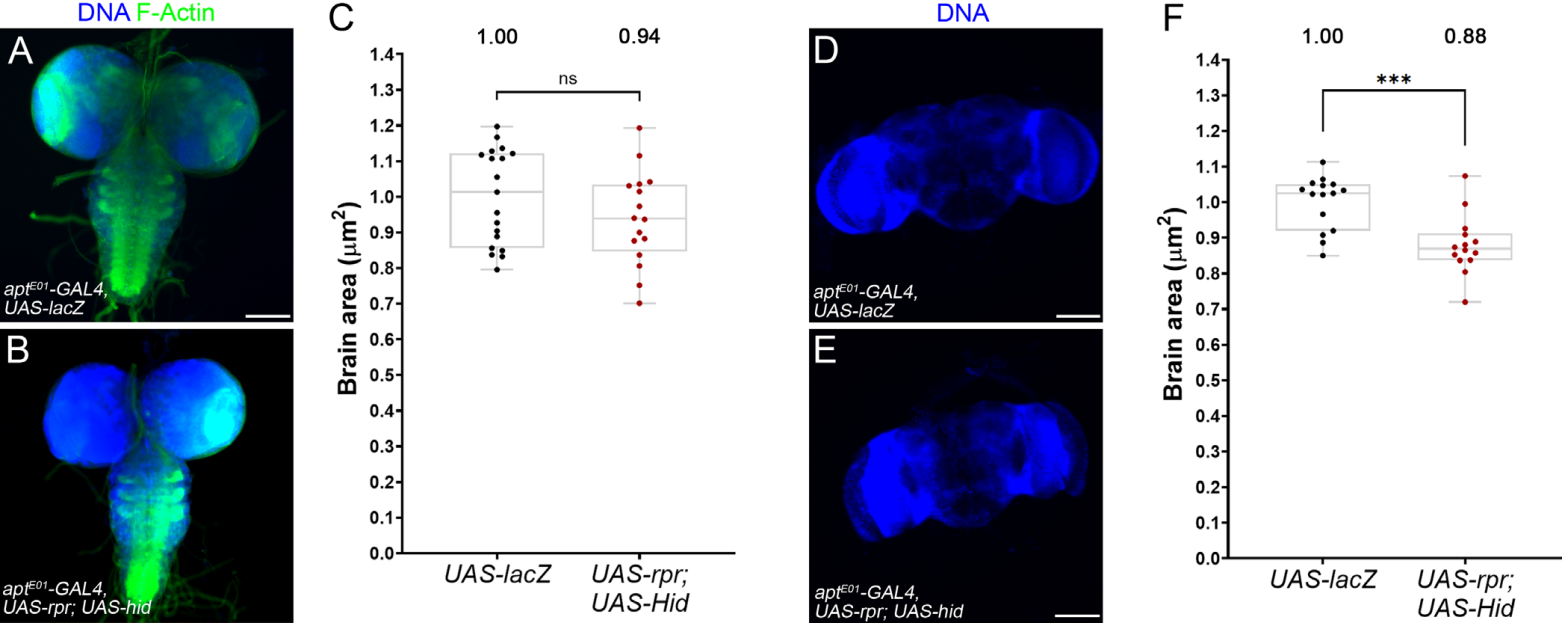
Ablation of perineurial glia affects adult brain size. (A, B, D, E) CNS preparations of third instar larva and adult brains of the indicated genotype stained for DNA using DAPI (blue), and F‐actin using Phalloidin (green). (C) Quantification of larval CNS area (*n* = 19 and 16). (F) Quantification of adult brain area (*n* = 15 and 14). Scale bars are 20 μm. Boxes and whiskers represent quartiles. Only female animals were analyzed. Unpaired Student's *t*‐test was performed. ns is not significant and *** corresponds to *p*‐value < 0.001.

To determine whether this phenotype reflected a generalized growth defect, we analyzed overall larval growth by measuring pupal size under PG ablation conditions. No significant differences were observed relative to control pupae (Figure S6A–C). In contrast, analysis of the adult female wing size revealed a modest reduction of approximately 4% in PG‐ablated animals compared with controls (Figure S6D–F). Together, these findings suggest that larval PG ablation primarily affects brain size.

### 2.4 Pleated Septate Junctions Are Altered by Perineurial Glial Ablation

Given that PG are part of the BBB, we wondered whether SPGs are affected upon PG ablation. To assess SPG integrity, we combined a GFP‐tagged allele of the pSJ component *Neurexin‐IV* (*Nrx‐IV:GFP*) with a newly generated SPG nuclear marker *moody‐mCherry* ^*nls*^, and ablated PG using the *apt* ^*E01*^ *‐GAL4* driver. During larval development, SPG in the brain lobes appeared morphologically normal, and pSJ organization was largely preserved following PG ablation (Figure 4A,C′). However, when analyzed at higher resolution in the Airyscan mode, pSJ strands were significantly wider in PG‐ablated brains compared with controls (Figure 4B,D,I).

**FIGURE 4:**
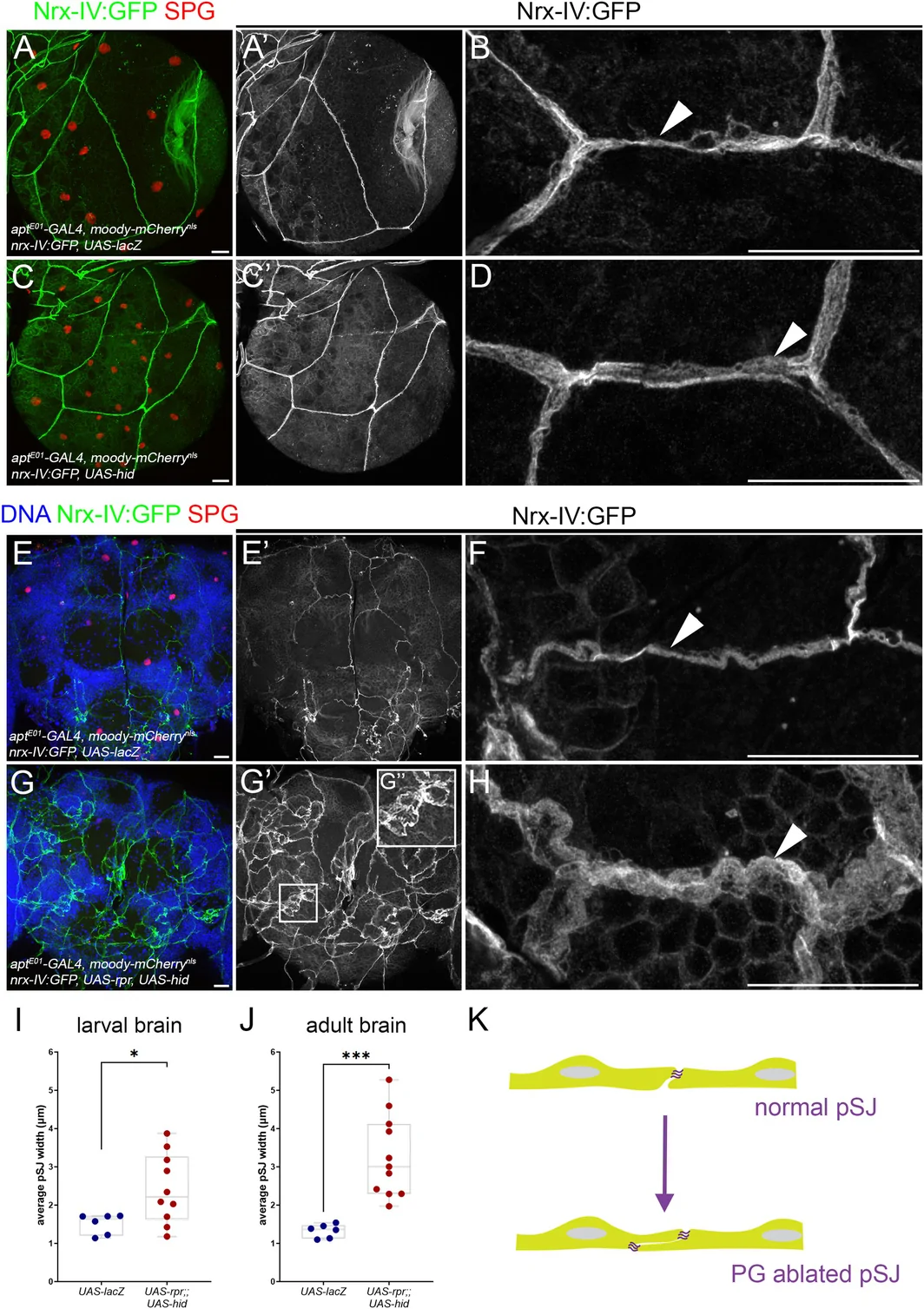
Pleated septate junctions are altered by perineurial glia ablation during larval development. (A–D) Larval brains of the genotypes (A, B) *apt* ^*E01*^ *‐GAL4; moody‐mCherry* ^*nls*^ (red), *Nrx‐IV:GFP* (green and gray); *UAS‐lacZ* (control) and (C, D) *apt* ^*E01*^ *‐GAL4; moody‐mCherry* ^*nls*^ (red), *Nrx‐IV:GFP* (green and gray); *UAS‐rpr;; UAS‐hid*. (B, D) Super resolution images. (E–H) Adult brains of the genotype (E–F) *apt* ^*E01*^ *‐GAL4; moody‐mCherry* ^*nls*^ (red), *Nrx‐IV:GFP* (green and gray); *UAS‐lacZ* (control) and (G–H) *apt* ^*E01*^ *‐GAL4; moody‐mCherry* ^*nls*^ (red), *Nrx‐IV:GFP* (green and gray); *UAS‐rpr;; UAS‐hid* stained with DAPI (blue). (G″) magnification of squared region in (G′). (F, H) Super resolution images. (I, J) Plot showing the average pSJ width in (I) larval and (J) adult brains in control (*UAS‐lacZ*) and ablated conditions (*UAS‐rpr;; UAS‐hid*). (K) Diagram representing pSJ strands in normal and PG‐ablated brains. Arrowheads point to septate junctions. Scale bars: 20 μm.

We next evaluated pSJ organization in the adult brain. Individual pSJ in PG‐ablated brains were severely disrupted compared with controls, displaying widened, frayed, and disorganized strands (Figure 4F,H,J). In addition, the signal of the *moody‐mCherry* ^*nls*^ reporter was diminished in PG‐ablated brains (Figure 4G). This might suggest that PGs activate *moody* expression which in turn is needed to control the organization of pSJ strands (Schwabe et al. 2005; Hatan et al. 2011; Babatz et al. 2018; Li et al. 2021). In agreement with this notion, we observed ectopic pSJ strands in brains subjected to PG ablation during development that created the appearance of fragmentation into cell patches lacking nuclei (Figure 4E–G′). Most likely, however, they may instead reflect the enlargement of the lateral cell–cell contact site, resulting in disrupted junctional pSJ strands (Figure 4K).

To determine when this excess pSJ phenotype arises, we analyzed the structure of pSJ in PG‐ablated brains during pupal development (Figure S7). Twenty four hours after puparium formation (hAPF), pSJ strands appeared wide and began to form large loop‐like structures (Figure S7A–D,K). As pupal development progressed, pSJs continued to widen and fray (Figure S7E–H), eventually forming shorter and highly disorganized strands by 72 hAPF (Figure S7I–K). Altogether, these results indicate that PG ablation affects SPG morphology that results in progressive splitting and fraying of pSJ strands during early pupal development.

The altered arrangement of pSJ strands observed in PG‐ablated brains could indicate impaired sealing of the BBB. To test this hypothesis, we injected 10 kDa dextran‐Texas Red into the abdomen of PG‐ablated flies and assessed dye penetration into the eye (Bainton et al. 2005; Pinsonneault et al. 2011). As a positive control for increased permeability, we also injected *moody* mutant flies (*moody* ^*ΔC17*^), which exhibit elevated paracellular permeability (Bainton et al. 2005). As expected, dye accumulation in the eye was significantly increased in *moody* mutants compared to control flies (Figure S8). In contrast, PG‐ablated animals showed permeability to 10 kDa dextran comparable to that of control flies (Figure S8). These results suggest that, despite severe alterations in pSJ morphology, paracellular BBB permeability is not markedly compromised in PG‐ablated animals.

### 2.5 Over‐Activation of FGF Signaling in Perineurial Glia Alters Pleated Septate Junction Organization

During development, proliferation of PG is regulated by the action of the fibroblast growth factor (FGF) and insulin (In) signaling (Franzdóttir et al. 2009; Avet‐Rochex et al. 2012). To determine the source of the FGF8‐like ligand Pyramus (Pyr), we used a *pyr‐T2A‐GAL4* line (Lee et al. 2018) crossed to a nuclear marker (*UAS‐redStinger*). In larval brains, we observed that at the surface of the brain only SPG nuclei expressed *pyr* (Figure 5A–A″). Next, we analyzed the expression of the FGF receptor, *heartless* (*htl*), using an endogenous reporter (*htl:mCherry*) (Irizarry and Stathopoulos 2015). This reporter showed that in addition to cortex glial cells, PG express Htl (Figure 5B,C″). This suggests that FGF‐mediated communication between SPG and PG regulates proliferation of the perineurial layer.

**FIGURE 5:**
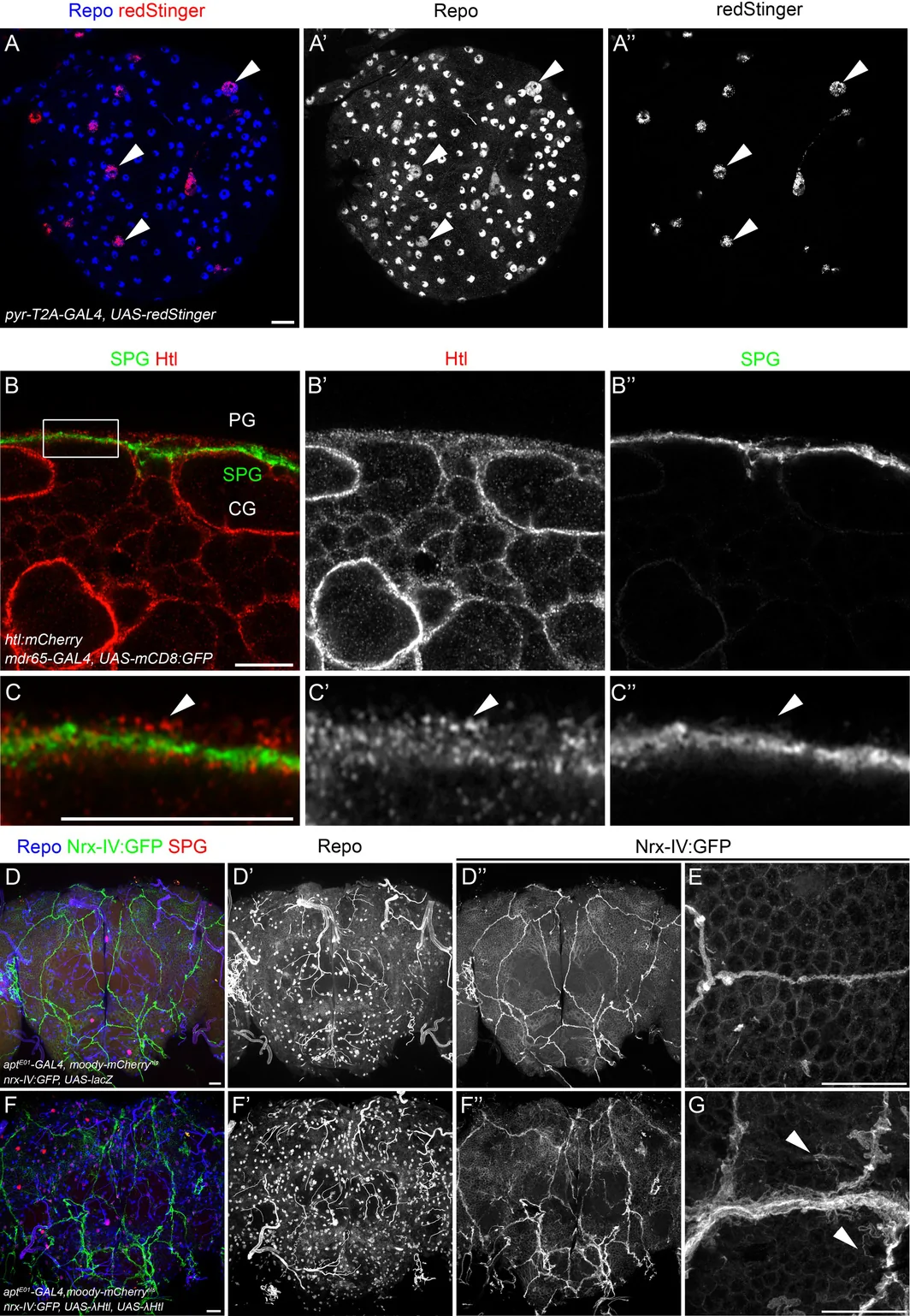
Increased FGF signaling in perineurial glia disrupts pleated septate junction structure. (A–A″) Immunostaining of a *pyr‐T2A‐GAL4, UAS‐redStinger* larval brain for Repo (blue). Arrowheads point to SPG (big glial nuclei) expressing *pyr*‐GAL4 reporter. (B–C″) Larval brain of the genotype *htl:mCherry; mdr65‐GAL4, UAS‐mCD8:GFP* animals. (C–C″) are enhanced super‐resolution images and arrowheads point to perineurial glia expression of Htl. (D–G) Adult brains stained for Repo (blue or gray) of the genotypes (D‐E) *apt* ^*E01*^ *‐GAL4; moody‐mCherry* ^*nls*^ (red), *Nrx‐IV:GFP* (green or gray); *UAS‐lacZ* (control) and (F–G) *apt* ^*E01*^ *‐GAL4; moody‐mCherry* ^*nls*^ (red), *Nrx‐IV:GFP* (green or gray); *UAS‐λHtl; UAS‐λHtl*. Note that tracheal labeling in blue channel is an autofluorescence artifact. (E, G) Super resolution images of pSJs. Arrowheads show detached pSJ strands. Scale bars: 20 μm. CG, Cortex glia, PG, perineurial glia; SPG, Subperineurial glia.

Given that reducing PG number by ablation resulted in remodeling of pSJ, we asked whether increasing the number of PG, by ectopic activation of the FGF signaling, could itself perturb the structure and organization of pSJ in SPG. To test this, we induced PG proliferation by expressing a constitutively active form of the FGF receptor (λHtl) and subsequently examined the structure of pSJ between SPG. Induced PG proliferation resulted in normal pSJ morphology in larval stages (Figure S9A–E), however, in adult brains we observed pronounced alterations in pSJ morphology, with junctional strands appearing widened and frayed, and in some cases detached from the main junctional network (see arrowheads in Figure 5D–G, Figure S9F). In contrast to PG ablation, ectopic PG proliferation only rarely led to the formation of ectopic pSJ strands (Figure 5F). These findings indicate that the precise regulation of FGF signaling and PG number is required to maintain SPG junctional integrity.

### 2.6 Ablation of Perineurial Glia Increases Sensitivity to Glucose‐Only Food

One of the principal roles attributed to PG is the carbohydrate transport across the BBB (Volkenhoff et al. 2015, 2018; McMullen et al. 2021). To assess the impact of PG ablation on carbohydrate transporter expression, we performed immunofluorescence analysis of the trehalose transporter Tret1. Under control conditions, Tret1 was robustly expressed in PG at the surface of the larval brain; however, this expression was significantly reduced following PG ablation using the *apt* ^*E01*^ *‐GAL4* driver (Figure 6A,B′,E and Figure S10). Similarly, analysis of the glucose and trehalose transporter Pippin using an HA‐tagged reporter line (*pippin‐3xHA*) (McMullen et al. 2021) revealed a marked reduction in the BBB upon PG ablation (Figure S11).

**FIGURE 6:**
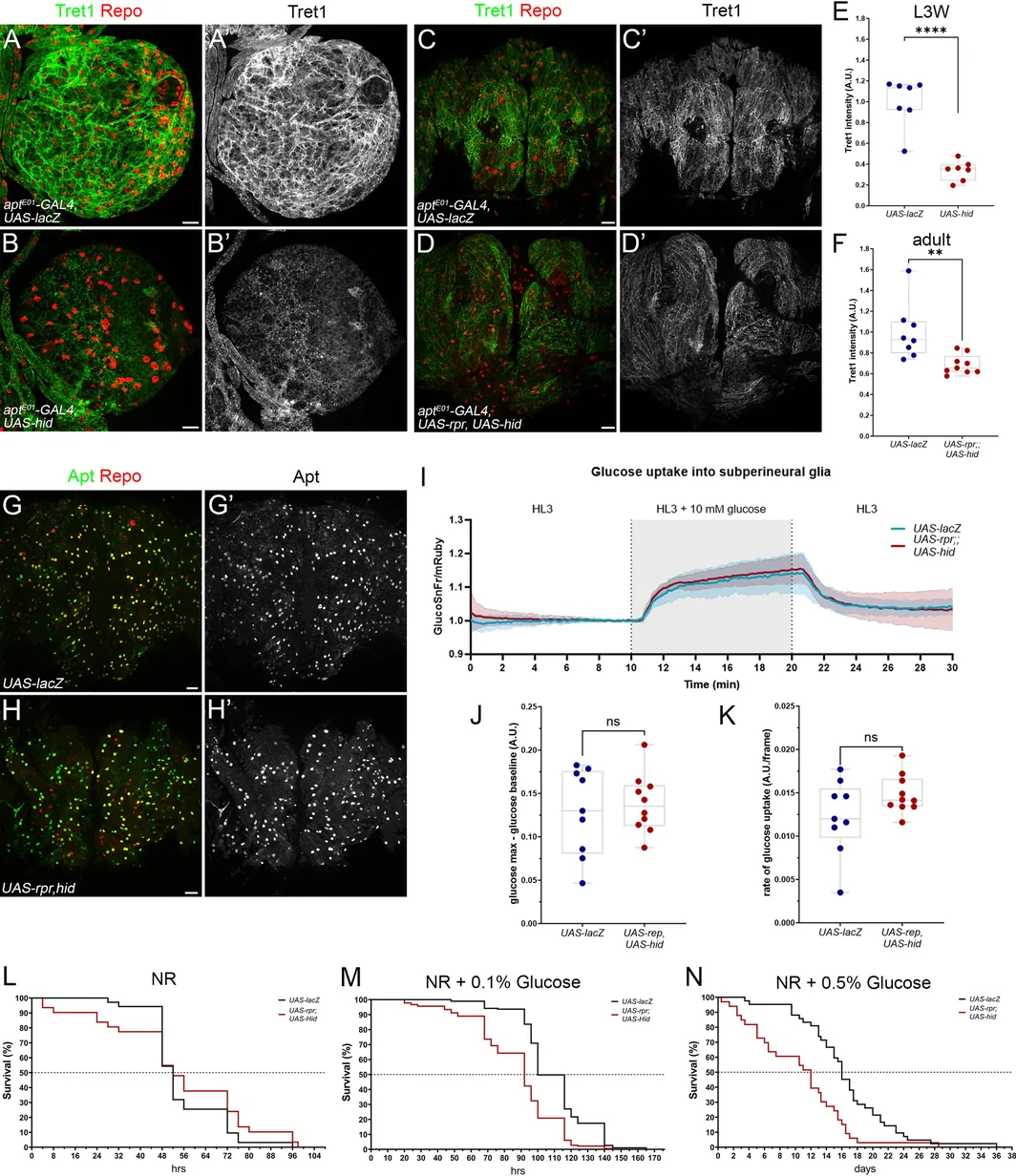
Perineurial glia ablation increases sensitivity to glucose‐only diet. (A, B′) Larval and (C, D′) adult brains stained for Tret1 (green or gray) and Repo (red). The following genotypes are used: (A, A′ C, C′) *apt* ^*E01*^ *‐GAL4; UAS‐lacZ* (control), (B, B′) *apt* ^*E01*^ *‐GAL4; UAS‐hid* and (D, D′) *apt* ^*E01*^ *‐GAL4; UAS‐rpr; UAS‐hid*. (E, F) Graphs showing Tret1 intensity quantification of (E) larval and (F) adult brains. Student's *t*‐test was performed. **** and ** are *p*‐value < 0.0001 and < 0.01, respectively. (G–H′) Adult brains stained for Apt (green or gray) and Repo (red). Genotypes are *apt* ^*E01*^ *‐GAL4; UAS‐lacZ* (control) and *apt* ^*E01*^ *‐GAL4; UAS‐rpr; UAS‐hid*. Scale bars are 20 μm. (I) Graph showing the fluorescence intensity ratio of the Glucose sensor and mRuby of *lexAop‐ iGlucoSnFR.mRuby2; apt* ^*E01*^ *‐GAL4; mdr65‐LexA; UAS‐lacZ* (blue) and *lexAop‐ iGlucoSnFR.mRuby2; apt* ^*E01*^ *‐GAL4; mdr65‐LexA; UAS‐rpr;;UAS‐hid* (red). Larval CNS were exposed to HL3 or HL3 with 10 mM glucose. Quantification of the (J) maximal glucose level and (K) the rate of glucose uptake (*N* = 3, *n* = 9). (L‐N) Animal lifespan of the genotypes *apt* ^*E01*^ *‐GAL4*; *UAS‐lacZ* (black) and *apt* ^*E01*^ *‐GAL4*; *UAS‐rpr; UAS‐hid* adult flies subjected to (L) complete nutrient restriction (NR, *n* = 33 and 30, *p*‐value = 0.3783), (M) NR supplemented with 0.1% glucose (*n* = 189 and 182, *p*‐value < 0.0001), and (N) NR supplemented with 0.5% glucose (*n* = 42 and 33, *p*‐value = 0.0002). Welch's *t*‐test and Log‐rank (Mantel‐Cox) test were performed. ns, not significant.

In adult brains, Tret1 was detected at the brain surface in both control and PG‐ablated conditions; however, its levels were also significantly reduced in PG‐ablated animals (Figure 6C,D′,F). To determine whether these glial cells correspond to PG, we performed co‐staining for Apt and Repo markers. In both control and PG‐ablated brains, we observed the presence of Apt/Repo‐positive glial cells at the brain surface, confirming that the perineurial layer is at least partially restored after developmental ablation. Together, these results suggest that the loss of PG during larval development leads to at least a transient reduction of carbohydrate transporter expression at the BBB.

The loss of Tret1 expression in the larval brain after PG ablation suggested a potential defect in carbohydrate transport across the BBB. To test this, we expressed the genetically encoded glucose sensor iGlucoSnFR.mRuby2 (Keller et al. 2019) in SPG using the LexA/lexAop system, while simultaneously inducing PG apoptosis via the GAL4/UAS system. Third instar larval brains were mounted in an ex vivo perfusion chamber (Volkenhoff et al. 2018) and glucose influx was quantified following perfusion with 10 mM glucose. Surprisingly, we observed that glucose influx in SPG was comparable between control and PG‐ablated brains (Figure 6I), with no significant differences in either the maximal glucose levels or uptake kinetics (Figure 6J,K). These results suggest that SPG can sustain glucose transport across the BBB in larvae despite PG ablation.

Given that glucose transport was not impaired by PG ablation in larvae, we hypothesized that PG loss may have metabolic consequences in adults. To test this, we subjected adult flies to nutrient restriction (NR) after eclosion and assessed survival. Under complete starvation, we observed that the median survival was identical for both control and PG‐ablated conditions (52 h, Figure 6L), suggesting that ablation of PG during larval development did not affect general starvation tolerance.

We next examined whether a glucose‐only diet would differentially affect survival. To this end, we supplemented the NR diet with different low concentrations of glucose (0.1% and 0.5%) and measured the median survival on the respective food. In general, the addition of glucose to the diet prolonged the median survival rate; however, PG‐ablated flies exhibited significantly reduced survival compared with controls under both conditions: 92 versus 100 h on 0.1% glucose (Figure 6M), and 12 versus 16 days on 0.5% glucose (Figure 6N). These results suggest that PG participate in brain carbohydrate uptake, revealing a potential long‐term metabolic vulnerability associated with disrupted PG function during development.

## 3 Discussion

The *Drosophila* blood–brain barrier (BBB) is a multilayered glial interface in which two distinct surface glial populations work together as gatekeepers between hemolymph and the nervous system. The perineurial glial cells (PG) form the outermost cellular layer and have been implicated in the uptake of sugars such as trehalose and glucose, whereas the subperineurial glia (SPG) establish the primary diffusion barrier by forming extensive pleated septate junctions (pSJs). Although this division of labor is well recognized, it remains incompletely understood how these two glial layers coordinate during development to match tissue growth, membrane expansion, and junctional stability.

To address this, we developed a PG‐specific genetic tool (*apt* ^*E01*^ *‐GAL4*) and used it to ablate PG during development by inducing apoptosis. Genetic cell ablation has been widely employed to successfully elucidate the function of distinct glial populations in *Drosophila* (Muthukumar et al. 2014; Stork et al. 2014; Coutinho‐Budd et al. 2017; Kottmeier et al. 2020; Pogodalla et al. 2021; Bajar et al. 2022; Beachum et al. 2025). Previously, PG were ablated, causing lethality in adults (Parkhurst et al. 2018). Parkhurst et al. used the *indy‐GAL4*; however, *indy* is expressed not only in PG but also in the fat body, midgut, and oenocytes (Knauf et al. 2002). Therefore, we cannot exclude the possibility that the reported lethality phenotype resulted, at least in part, from the ablation of these additional cell types rather than from PG loss alone.

Using this genetic ablation approach, we uncovered an unexpected temporal separation of PG requirements. Despite the loss of most PG, glucose influx into SPG was largely preserved during larval stages, whereas pSJ organization became progressively disrupted during pupal development, culminating in widened, frayed, and ectopic junctional strands. Notably, some PG escaped the genetic cell death induction and possibly divide during pupal stages. However, these PG still render the adult fly with an increased sensitivity to a glucose‐only dietary restriction. Although the origin of this sensitivity is unknown, it correlates with a reduction in Tret1 levels and is likely that the efficiency of carbohydrate transport is impaired in adult animals after larval PG ablation.

The viability and apparently normal morphology of PG‐ablated larvae suggest that PG are largely dispensable during early development—likely because key aspects of their function can be compensated by SPG. The preserved glucose influx across the larval BBB in the absence of PG supports this notion and indicates that glucose delivery to SPG can be maintained even when PG‐associated carbohydrate transporters are lost. This functional resilience may reflect redundancy in glucose transport mechanisms (Volkenhoff et al. 2018) or compensatory regulation within SPG (Hertenstein et al. 2021).

Given that trehalose is the principal circulating carbohydrate in insects, we cannot exclude the possibility that its transport and metabolism are affected by the loss of PG. The high expression of the trehalose transporters Tret1 and Pippin in PG (Volkenhoff et al. 2015; McMullen et al. 2021) supports the idea that trehalose metabolism represents a major functional role of PG at the BBB. Moreover, previous work has shown that exposure of brains to trehalose does not increase glucose levels in SPG (Volkenhoff et al. 2018), suggesting that PG may metabolize trehalose and deliver a metabolic intermediate such as pyruvate or lactate to the SPG. Notably, flies can survive without synthesizing trehalose (Matsuda et al. 2015; Matsushita and Nishimura 2020), suggesting that this metabolic function of PG, while physiologically relevant, may be largely dispensable under standard conditions. However, our experiments do not allow us to conclude that PG functions are nonessential. The PG that remain after ablation with the *apt* ^*E01*^ *‐GAL4* driver could partially preserve PG‐dependent functions in the brain.

We observed that in the central brain and vnc there are *apt* ^*E01*^‐negative PG, suggesting that this subpopulation may serve as a reservoir of PG. Previous studies have shown that PG proliferate predominantly during larval development in both the central and peripheral nervous system (Awasaki et al. 2008; Avet‐Rochex et al. 2012; Franzdóttir et al. 2009; Matzat et al. 2015; von Hilchen et al. 2013). Based on this evidence, we favor the hypothesis that, in our experimental paradigm, a*pt* ^*E01*^‐negative PG proliferate and repopulate the brain during metamorphosis. However, these cells show reduced expression of the *Tret1*, suggesting that they may provide diminished metabolic support to the central nervous system.

Our findings indicate that PG dynamics play an important role in shaping the development and organization of SPG pSJs. The widened and frayed morphology of pSJs in PG‐ablated brains closely resembles the phenotypes described in mutants affecting the GPCR/Moody and PKA signaling (Schwabe et al. 2005; Babatz et al. 2018; Li et al. 2021). This notion is supported by the reduced expression of the *moody* expression reporter in PG‐ablated brains; however, the pSJ phenotype observed may be the consequence of alterations in several signal pathways. Importantly, loss of moody affects the establishment of pSJs during embryogenesis; meanwhile, PG ablation seems to alter the structure of pSJs mainly during early pupal development.

Notably, SPG may also directly regulate PG development. Pyr/FGF8 is exclusively expressed in SPG; however, its activity has been shown to be required for proper PG proliferation (Franzdóttir et al. 2009; Avet‐Rochex et al. 2012). This interaction raises the possibility of coordinated regulation between these glial cells during development. Overactivation of FGF signaling in PG increases PG proliferation but also disrupts pSJ structure in SPG, suggesting that a balance between PG number and SPG development may be tightly controlled (Figure 7). Nevertheless, how PG signal back to SPG to regulate pSJ formation during development remains unknown.

**FIGURE 7:**
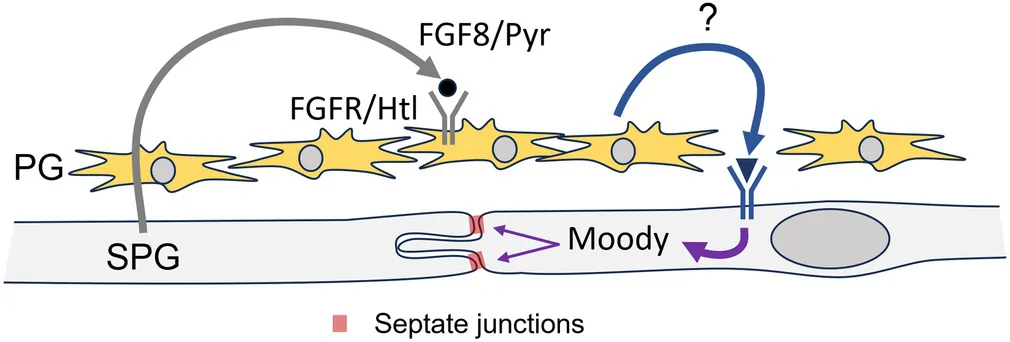
Working model. The establishment of the BBB is tightly controlled by SPG and PG interactions. SPG express *pyr*, which regulates PG proliferation during development. Meanwhile, the proper number of PG is required to signal back to the SPG, thereby influencing pSJ formation and *moody* expression. Moody/GPCR signaling may therefore represent one of the pathways involved in the response of SPG to PG‐derived signals.

Importantly, our work has several limitations. A comprehensive understanding of PG function will require complete and temporally controlled ablation of these cells throughout the brain. Such an approach would allow the distinct developmental and physiological roles of PG to be disentangled. For example, PG may be required for pSJ maturation during development, whereas their metabolic support may be more critical for maintaining BBB function in adults. Additionally, it is unknown whether the phenotypes observed in the central brain are also recapitulated in the vnc or the blood‐retinal barrier.

Further studies will therefore be necessary to define the specific contributions of PG to BBB development, maturation, and physiological function. The development of new genetic tools enabling complete and temporally controlled PG ablation, either during development or specifically in adulthood, will provide a more definitive assessment of PG function and their coordination with SPG in establishing and maintaining BBB integrity.

## 4 Material and Methods

### 4.1 *Drosophila* Genetics

All *Drosophila* work was conducted according to standard procedures. Fly stocks were kept in standard food at room temperature, while experimental crosses were maintained at 25°C. The following stocks were used: *46F‐GAL4* (Brink et al. 2012), *GMR49G07‐GAL4* (Jenett et al. 2012), *mdr65* ^*GMR54C07*^ *‐LexA* (BDSC #94728), *pyr* ^*CR01744*^ *‐T2A‐GAL4* (BDSC # 91292), *Tret‐1‐1‐Stinger* (Hertenstein et al. 2021), *Tret1‐GAL80* (Winkler et al. 2021), *htl:mCherry* (Irizarry and Stathopoulos 2015), *pippin‐3xHA* (McMullen et al. 2021), *LanB1:GFP* (VDRC v318180) (Sarov et al. 2016), *Nrx‐IV:GFP* (Edenfeld et al. 2006), *UAS‐lam:GFP*, *UAS‐mCD8:mCherry*, *UAS‐mCD8:GFP*, *UAS‐H2B:mRFP*, *UAS‐Stinger* (BDSC #90920), *UAS‐redStinger*, *UAS‐lacZ*, *UAS‐rpr*, *UAS‐hid*, *UAS‐λHtl* (BDSC #5367), *lexAop‐iGlucoSnFR.mRuby2* (BDSC #82993) (Keller et al. 2019), *moody* ^*ΔC17*^ (Bainton et al. 2005; Schwabe et al. 2005).

### 4.2 Generation of Transgenic Lines

The *apt* ^*E01*^ enhancer was amplified from *pBPGUw‐GMR49G07‐mCherry* using the following primers: *5′‐CACCCCGAAGAACGCGTTGAGTAAATGCA‐3′* and *5′‐TCCAGATCGGTGGCCGAATACTGTT‐3′*. The PCR product was cloned into pENTR/D‐TOPO and subsequently subcloned into *pBPGUw* using the Gateway LR recombination reaction to generate *pBPGUw‐apt* ^*E01*^. Using a standard germline transformation protocol, this plasmid was injected into the attP40 landing site to generate *apt* ^*E01*^ *‐GAL4* flies.

To generate a nuclear SPG marker, *moody‐mCherry* ^*nls*^ construct, we used *pENTR‐moody* plasmid containing 2.4 kb of *moody* promoter to subclone this into the pVanGlow‐RR‐DSCP (addgene Plasmid #83339) using the Gateway LR recombination reaction. The resulting plasmid was injected into *attP40* and *attP2* landing sites.

### 4.3 Immunofluorescence

Third instar wandering larval, pupal, and adult brains were dissected in 1× PBS and fixed in 4% formaldehyde in 1× PBS for 20 min. Brains were washed in 0.3% Triton X‐100 in 1× PBS (PBT) for 5 min. Samples were blocked in 10% normal goat serum in PBT. Primary antibodies: rabbit anti‐Apt (1:150) (Liu et al. 2003), mouse anti‐β‐Galactosidase (1:20, 40‐1a DSHB), mouse anti‐Repo (1:20, 8D12 DSHB), rabbit anti‐HA (1:1000 Cell Signaling), guinea pig anti‐Tret1 (1:200) (Volkenhoff et al. 2015). AlexaFluor conjugated secondary antibodies (ThermoFisher) were used at a concentration of 1:200 in PBT, 10% goat serum. DAPI and phalloidin‐AlexaFluor647 were incubated at 1:1000 and 1:200 dilution, respectively. Imaging was performed using confocal microscopes (Leica TCS SP8, Zeiss LSM 710, or LSM 880 with Airyscan) and a Zeiss Axio Zoom.v16 fluorescent stereomicroscope.

Images, diagrams, and figures were assembled using Fiji (Image J, NIH), Adobe Photoshop CC, and Adobe Illustrator CC.

### 4.4 BBB Permeability Assay

To assess BBB permeability, female adult flies were injected in the abdomen with approximately 70 nL of 12.5 mg/mL 10 kDa dextran‐Texas red (#D1863, Invitrogen) using a Nanoject II Auto‐Nanoliter Injector (Drummond Scientific). Flies were allowed to recover at room temperature and then imaged in a Leica fluorescent stereomicroscope. Mean fluorescence intensity within a circular ROI in the eye was measured and plotted.

### 4.5 Quantifications and Data Analysis

The volume of pupae was estimated using the ellipsoid volume formula 4/3π(L/2)(d/2)^2^ (L, length; d, diameter) (Layalle et al. 2008). To analyze wing size, adult flies, 2–3 days after eclosion, were fixed in 95% ethanol for 24 h. Next, wings were mounted in a 1:1 lactic acid: ethanol solution. Fiji (Image J, NIH) was used to measure the area of each wing. Only female animals were analyzed. Pleated septate junctions were quantified by measuring their area and dividing by the total length of the pSJ, obtaining the average width. Plots showing quartiles and statistical analyses were performed using GraphPad Prism 10.

### 4.6 Measurement of Glucose Uptake

Third instar larval brains were dissected in HL3 buffer without added trehalose (70 mM NaCl, 5 mM KCl, 1.5 mM CaCl_2_·2H_2_O, 20 mM MgCl_2_·6H_2_O, 10 mM NaHCO_3_, 115 mM sucrose, 5 mM HEPES, pH = 7.2). Brains were then mounted on coverslips coated with poly‐L‐lysine (Sigma‐Aldrich, USA) and mounted in a custom‐made flow‐through perfusion chamber (McMullen et al. 2022). The chamber containing the samples was placed on a Zeiss Axio.ImagerZ2 epifluorescence microscope (Carl Zeiss Microscopy GmbH, Germany) and imaged using Zeiss W Plan‐Apochromat 20× 0.5 objective (Carl Zeiss Microscopy GmbH, Germany). Buffer flow was generated using a mini‐peristaltic pump at a flow rate of 7.5 mL/min (Cole‐Parmer, USA). Samples were illuminated with an X‐Cite Xylis white‐light LED (Excelitas Technologies Corp., USA). The iGlucoSnFR glucose sensor was excited using a 470/40 nm excitation filter (F49‐470, Analysentechnik, Germany) while the reference fluorophore mRuby was excited using a 572/35 nm excitation filter (F49‐572 Analysentechnik, Germany). Emission was recorded using a dual bandpass filter (F57‐019, Analysentechnik, Germany) in combination with a dichroic beam splitter (F58‐019, Analysentechnik, Germany). Images were acquired using an Axiocam 705 mono camera (Carl Zeiss Microscopy GmbH, Germany) at a frame rate of 6 frames per minute. Experiments were carried out in three biological replicates; each replicate including control and PG‐ablated brains mounted on the same slide. Initially, HL3 buffer without trehalose was perfused for 10 min to establish a stable baseline fluorescent signal, followed by perfusion with HL3 buffer without trehalose supplied with 10 mM glucose for 10 min, and lastly return to HL3 buffer without trehalose for another 10 min. Data analysis was performed by generating a ROI including the VNC and calculating mean gray values for both fluorescent channels. The ratio of iGlucoSnFR to mRuby fluorescence intensity (mean gray values) was plotted over time. The glucose uptake and rate of glucose uptake were calculated as previously described (McMullen et al. 2022).

## Author Contributions

E.G.C., S.S. and C.K. conceptualized and designed experiments. E.G.C., E.N., M.C.‐R., N.V.S., M.R. and J.S.‐T. performed experiments. E.G.C. and C.K. wrote the original manuscript.

## Funding

The authors declare that financial support was received for the research, authorship, and/or publication of this article. E.G.C. was supported by ANID‐FONDECYT Iniciación en Investigación 2023 No. 11230539. This work was also supported by the Deutsche Forschungsgemeinschaft through funds to C.K. (SFB 1348‐B5, Kl588/29) and S.S. (SCHI1380/6‐1).

## Conflicts of Interest

The authors declare no conflicts of interest.

## Supporting information


**Figure S1:** Expression pattern of *46F‐GAL4* driver. (A–F) Larval CNS of the genotype *46F‐GAL4*; *UAS‐mCD8:GFP, UAS‐H2B:mRFP* stained for GFP (green), RFP (red), and the pan‐glial marker Repo (blue). Arrow points expression in cortex glia in the optic lobes. Arrowhead marks subperineurial glial nucleus.

**Figure S2:** Expression pattern of *GMR49G07‐GAL4* driver. (A–F) Larval CNS of genotype *GMR49G07‐GAL4; UAS‐mCD8:GFP, UAS‐H2B:mRFP* stained for GFP (green), RFP (red), Repo (magenta) and Apontic (Apt, blue). Arrowheads points to tracheal glial nucleus (RFP+/Apt+/Repo−).

**Figure S3:** Expression pattern of *apt* ^*E01*^ *‐GAL4* driver. (A–A″) Larval brain lobe of the genotype *apt* ^*E01*^ *‐GAL4*; *UAS‐Stinger* stained for GFP (green), Apt (red), and Repo (blue). Arrowheads indicate perineurial glial nuclei that do not express the driver (Stinger−/Apt+/Repo+). (B–B″) Larval CNS of *apt* ^*E01*^ *‐GAL4, Tret1‐Stinger, moody‐mCherry* ^*nls*^; *UAS‐lacZ* immunostained for β‐Galactosidase (red). Arrowheads indicate perineurial glia that express high levels of *apt* ^*E01*^ *‐GAL4* driver in the optic lobe. Scale bars are (A–A″) 20 μm, (B–B″) 50 μm.

**Figure S4:** Ablation of perineurial glia in the CNS. (A–B′) Immunofluorescence of larval CNS of the genotypes: (A, A′) *apt* ^*E01*^ *‐GAL4, UAS‐mCD8:mCherry* (red)*; UAS‐lacZ* (control), and (B, B′) *apt* ^*E01*^ *‐GAL4, UAS‐mCD8:mCherry* (red); *UAS‐hid* and stained for Tret1 (green), mCherry (red), and Repo (blue). Scale bars are 100 μm. (C) Quantification of the number of PG (Stinger‐positive) in *apt* ^*E01*^ *‐GAL4, Tret1‐Stinger, UAS‐lacZ* and *apt* ^*E01*^ *‐GAL4, Tret1‐Stinger, UAS‐rpr, UAS‐hid* larval brains. Unpaired Student's t test was performed. **** corresponds to *p*‐value < 0.0001.

**Figure S5:** Some perineurial glia remain in the CNS after genetic ablation. Larval CNS of the genotypes (A–A″, C‐C″) *apt* ^*E01*^ *‐GAL4; UAS‐lacZ* and (B–B″, D–D″) *apt* ^*E01*^ *‐GAL4; UAS‐rpr;; UAS‐hid*, and immunostained for Apt (green) and Repo (red). Arrowheads show remaining perineurial glial nuclei (Apt+/Repo+) in ablated CNS. Scale bar is 20 μm.

**Figure S6:** Growth of PG ablated animal is not affected. (A, B) Images of pupae of the following genotypes: (A) *apt* ^*E01*^ *‐GAL4; UAS‐lacZ*, and (B) *apt* ^*E01*^ *‐GAL4; UAS‐rpr;; UAS‐hid*. (C) Quantification of pupal volume of female animals (*n* = 47 and 50). (D, E) Wings of adult female flies of genotypes: (D) *apt* ^*E01*^ *‐GAL4; UAS‐lacZ* (control), and (E) *apt* ^*E01*^ *‐GAL4; UAS‐rpr;; UAS‐hid*. (F) Quantification of wing size (*n* = 33 and 34). Scale bars are 1 mm. Unpaired Student's t test was performed. ns is not significant and *** corresponds to *p*‐value < 0.001. Only female animals were analyzed.

**Figure S7:** Pleated septate junctions during pupal development. (A–K) Super resolution images of pSJs of (A, C, E, G, I) control genotype *apt* ^*E01*^ *‐GAL4; Nrx‐IV:GFP* (gray); *UAS‐lacZ* and (B, D, F, H, J) ablated animals expressing *UAS‐rpr;; UAS‐hid* at (A, B) third instar wandering (L3W), (C, D) 24 hAPF, (E, F) 30 h APF, (G, H) 48 h APF, and (I, J) 72 h APF. (K) Graph showing the average width of pSJs at different timepoints. Two‐way ANOVA and Bonferroni's multiple comparisons test were performed. **** is *p*‐value < 0.0001 and ns is non‐significant. (L) Schematic representation of the progression of pSJ phenotype after perineurial glia ablation. Scale bars: 20 μm.

**Figure S8:** Paracellular BBB permeability is not affected by perineurial glia ablation. (A–C) Images of the eye of flies of the following genotypes: (A) *apt* ^*E01*^ *‐GAL4; UAS‐lacZ* (control), (B) *apt* ^*E01*^ *‐GAL4; UAS‐rpr; UAS‐hid*, and (C) *moody* ^*ΔC17*^ mutant, injected with 10 kDa dextran‐Texas red in the abdomen. (D) Quantification of the dextran intensity in the eye. One‐way ANOVA followed by Dunnett's test were performed. **** is *p*‐value < 0.0001. Only female animals were analyzed.

**Figure S9:** Increased FGF signaling in perineurial glia does not disrupt pleated septate junction structure in larval brains. (A–D) Larval brains stained for Repo (red) and Apt (blue) of the genotypes *apt* ^*E01*^ *‐GAL4*, *Nrx‐IV:GFP* (green or gray); *UAS‐lacZ* (control) and (F, G) *apt* ^*E01*^ *‐GAL4, Nrx‐IV:GFP* (green or gray); *UAS‐λHtl; UAS‐λHtl*. Scale bars are 20 μm. (E, F) Quantifications of the average pSJ width in (E) larval and (F) adult brains. Student's *t*‐test was performed. ** is *p*‐value < 0.01 and ns is non‐significant.

**Figure S10:** Tret1 expression after perineurial glia ablation. (A, B) Orthogonal view of larval brains of the genotypes (A) *apt* ^*E01*^ *‐GAL4; UAS‐lacZ* and (B) *apt* ^*E01*^ *‐GAL4; UAS‐hid* larval brains stained for Tret1 (green) and Repo (red). Scale bars are 20 μm.

**Figure S11:** Carbohydrate transporter expression in perineurial glia ablated brains. (A‐B″) Larval brains of the following genotypes: (A) *apt* ^*E01*^ *‐GAL4; pippin‐3xHA*; *UAS‐lacZ*, and (B) *apt* ^*E01*^ *‐GAL4; pippin‐3xHA*; *UAS‐rpr; UAS‐hid* stained for Tret1 (green or gray), HA (red or gray) and Repo (blue). Scale bar is 20 μm. (C) Quantification of Pippin‐3xHA intensity. Student's *t*‐test was performed. **** is *p*‐value < 0.0001.

## Data Availability

The original contributions presented in the study are included in the article/Supporting Information further inquiries can be directed to the corresponding authors.
